# Thalamocortical functional connectivity and rapid antidepressant and antisuicidal effects of low-dose ketamine infusion among patients with treatment-resistant depression

**DOI:** 10.1038/s41380-024-02640-3

**Published:** 2024-07-06

**Authors:** Pei-Chi Tu, Wan-Chen Chang, Tung-Ping Su, Wei-Chen Lin, Cheng-Ta Li, Ya-Mei Bai, Shih-Jen Tsai, Mu-Hong Chen

**Affiliations:** 1 Department of Psychiatry, Taipei Veterans General Hospital, Taipei, Taiwan; 2 Division of Psychiatry, School of Medicine, College of Medicine, National Yang Ming Chiao Tung University, Taipei, Taiwan; 3 Department of Medical Research, Taipei Veterans General Hospital, Taipei, Taiwan; 4 Institute of Philosophy of Mind and Cognition, National Yang Ming Chiao Tung University, Taipei, Taiwan; 5 Department of biomedical engineering, National Yang Ming Chiao Tung University, Taipei, Taiwan; 6 Institute of Brain Science, National Yang Ming Chiao Tung University, Taipei, Taiwan; 7 Department of Psychiatry, General Cheng Hsin Hospital, Taipei, Taiwan

**Keywords:** Neuroscience, Depression

## Abstract

Previous studies have shown an association between the thalamocortical dysconnectivity and treatment-resistant depression (TRD). Whether a single subanesthetic dose of ketamine may change thalamocortical connectivity among patients with TRD is unclear. Whether these changes in thalamocortical connectivity is associated with the antidepressant and antisuicidal effects of ketamine treatment is also unclear. Two resting-state functional MRIs were collected in two clinical trials of 48 patients with TRD (clinical trial 1; 32 receiving ketamine, 16 receiving a normal saline placebo) and 48 patients with TRD and strong suicidal ideation (clinical trial 2; 24 receiving ketamine, 24 receiving midazolam), respectively. All participants underwent rs-fMRI before and 3 days after infusion. Seed-based functional connectivity (FC) was analyzed in the left/right thalamus. FCs between the bilateral thalamus and right middle frontal cortex (BA46) and between the left thalamus and left anterior paracingulate gyrus (BA8) increased among patients in the ketamine group in clinical trials 1 and 2, respectively. FCs between the right thalamus and bilateral frontal pole (BA9) and between the right thalamus and left rostral paracingulate gyrus (BA10) decreased among patients in the ketamine group in clinical trials 1 and 2, respectively. However, the associations between those FC changes and clinical symptom changes did not survive statistical significance after multiple comparison corrections. Whether ketamine-related changes in thalamocortical connectivity may be associated with ketamine’s antidepressant and antisuicidal effects would need further investigation. Clinical trials registration: UMIN Clinical Trials Registry (UMIN-CTR): Registration number: UMIN000016985 and UMIN000033916.

## Introduction

Major depressive disorder with insufficient response to at least two trials of traditional antidepressants is commonly referred to as treatment-resistant depression (TRD) [1, 2]. TRD is linked to a high relapse rate, suicidal thoughts and attempts, functional impairment, and a poor quality of life [1, 2]. Evidence has shown that by selectively binding to and inhibiting N-methyl-D-aspartate receptors (NMDARs) on γ-aminobutyric acid interneurons, ketamine—an NMDAR antagonist—increases synaptogenesis and brain-derived neurotrophic factor (BDNF) levels, ultimately producing antidepressant and antisuicidal effects [3, 4]. An international pooled patient-level meta-analysis of 720 patients with TRD demonstrated the robust main effects of a single ketamine infusion on acute and postacute depression severity [5]. A meta-analysis of 167 patients with TRD, bipolar depression, or posttraumatic stress disorder revealed that ketamine treatment rapidly (within 1 day) and significantly reduced suicidal ideation both in terms of clinician-rated and self-reported outcome measures [6]. Effect sizes were moderate to large (Cohen’s *d* = 0.48–0.85) at all time points following the infusion (days 1–7) [6].

Thalamus plays a crucial role in cortical sensory transmission [7]. Previous studies have discovered the thalamic engagement in the dynamic modulation of cortical activity in attention, executive and emotional control, and perceptual decision-making [7, 8]. TRD is associated with disrupted functional connectivity (FC) mainly in thalamocortical circuits [9, 10]. Lui et al. revealed that patients with TRD exhibited disrupted FC mainly in bilateral prefrontal areas and thalamus areas; by contrast, patients with non-TRD exhibited disrupted FC mainly in the bilateral anterior cingulate cortex, amygdala, hippocampus, and insula [9]. Kong et al. demonstrated that severe depression was associated with reduced FC between the thalamus and bilateral middle frontal cortex and with increased FC between the thalamus and right medial frontal cortex [10]. Furthermore, suicidal ideation and behaviors are associated with thalamocortical dysconnectivity [11–13]. A cross-sectional MRI study demonstrated that suicidal ideation was associated with structural alterations in regions of the salience network and thalamocortical circuit among patients with depression [11]. Zhang et al. showed that patients who attempted suicide exhibited significantly lower FC between the frontal cortex and bilateral thalamus than did those with no history of attempted suicide [12].

Increasing evidence suggests that the modulation of thalamocortical connectivity is associated with electroconvulsive therapy (ECT)-related antidepressant effects among patients with TRD [14, 15]. Takamiya et al. identified the FC regions associated with depressive symptom improvement following ECT, including the thalamus and sensorimotor cortex, by using data-driven multivoxel pattern analysis [15]. In a mouse study, Miller et al. indicated a role of the thalamocortical circuit, particularly the medial dorsal thalamus–medial prefrontal cortex (PFC) circuit, in the antidepressant effect related to the antagonism of the GluN2B-containing NMDARs [16]. Amat-Foraster et al. demonstrated that a subanesthetic dose of ketamine enhanced the discharge rates of principal neurons in the medial PFC and ventromedial thalamus in Wistar rats [17]. The short-lasting increase in thalamocortical activity was likely associated with the rapid psychotomimetic and antidepressant effects of ketamine [17]. Evidence has suggested the thalamus as a potential biomarker of the antidepressant effects of low-dose ketamine [18]. An open-labeled neuroimaging clinical trial of 38 patients with treatment-resistant bipolar depression demonstrated that increased global functional connectivity density was mainly located in the bilateral thalamus and anterior cingulate cortex [19]. Siegel et al discovered that medial thalamus played an important role in the sustained antidepressant effect of ketamine infusion [20]. However, whether the rapid antidepressant and antisuicidal effects of ketamine are associated with the thalamocortical FC changes among patients with TRD requires further investigation.

In the current study, we investigated thalamocortical connectivity in two randomized, double-blind, placebo-controlled trial samples, namely TRD patients in clinical trial 1 and TRD patients with strong suicidal ideation in clinical trial 2. Each participant underwent rs-fMRI before and 3 days after ketamine infusion. We hypothesized that the rapid antidepressant and antisuicidal effects of ketamine are associated with changes in thalamocortical connectivity among patients with TRD.

## Methods

### Participants

Our study team conducted two neuroimaging randomized, double-blind, placebo-control clinical trials of low-dose ketamine infusion in patients with TRD between 2012 and 2021 (Supplementary table 1, Supplementary figures 1 and 2) [21, 22]. The detailed study design, patient enrollment and clinical results of our study have been published [21, 22]. Briefly speaking, in clinical trial 1, 48 patients with TRD were randomly administrated with a single infusion of 0.5 mg/kg or 0.2 mg/kg ketamine or normal saline; in clinical trial 2, 48 patients with TRD and strong suicidal ideation (SI) were randomized to two groups receiving a single infusion of either 0.5 mg/kg ketamine or 0.045 mg/kg midazolam. In both clinical trials, TRD was defined as a major depressive disorder with poor or unsatisfactory response to at least two different antidepressants administered at an adequate dosage and for an adequate treatment duration in the current episode [21, 22]. The strong SI was defined by the scores of ≥4 at the MADRS item 10 in clinical trial 2 [22, 23]. Degrees of treatment refractoriness were measured by a points-based staging model, the Maudsley staging method (MSM) [24]. Depressive symptoms were examined using the 17-item Hamilton Depression Rating Scale (HDRS) and Montgomery-Asberg Depression Rating Scale (MADRS) immediately prior to infusion, at 40, 80, and 240 min postinfusion, and sequentially, on Days 2, 3, 5, 7 and 14 postinfusion both in clinical trials 1 and 2 [21, 22]. In addition, the Positive and Negative Suicide Ideation Inventory (PANSI) was self-reported at baseline, at 240 min postinfusion, and sequentially on Days 2, 3, and 7 postinfusion in clinical trial 2 [25]. The PANSI combines risk (negative suicide ideation, NSI) and protective (positive ideation, PI) factors to evaluate individual suicidal ideation [25]. In clinical trial 1, infusion-related dissociation symptoms were measured at baseline and 40-min postinfusion using the Brief Psychiatric Rating Scale positive subscale [26, 27]; in clinical trial 2, infusion-related dissociation symptoms were measured at baseline and 40-min postinfusion using the Clinician-Administered Dissociative States Scale [28, 29]. Baseline clinical characteristics, including body mass index (BMI), duration of illness, and history of suicidal attempt, and psychiatric comorbidities, were assessed. Exclusion criteria included major medical or neurological diseases or a history of alcohol or substance use disorders in both clinical trials. Furthermore, in order to elucidate the neurobiological effect of low-dose ketamine infusion, all participants underwent MRI prior to infusion and on Day 3 postinfusion. This study was conducted in accordance with the Declaration of Helsinki and was approved by the Institutional Review Board of Taipei Veterans General Hospital. All participants gave their written informed consent. Clinical trials registration: UMIN Clinical Trials Registry (UMIN-CTR): Registration number: UMIN000016985 and UMIN000033916.

### MRI image acquisition

MRI images were acquired using a 3.0 T Discovery MR750 (GE Healthcare) MRI scanner in clinical trial 1 and a 3.0 T SIGNA (GE Healthcare) PET/MR scanner in clinical trial 2. Head stabilization was achieved through cushioning, and all participants wore earplugs (29 dB rating) to attenuate noise. Automated shimming procedures were performed, and scout images were obtained. Resting-state functional images were collected using a gradient echo T2* weighted sequence (repetition time [TR]/echo time [TE]/flip angle = 2500 ms/30 ms/90°). Forty-seven contiguous horizontal slices parallel to the intercommissural plane (voxel size: 3.5 × 3.5 × 3.5 mm^3^) were acquired with an interleaved ascending slice order. These slices covering the cerebrum, cerebellum, and brainstem were acquired. During functional scanning, the participants were instructed to remain awake with their eyes open (each scan lasted 8 min and 24 s across 200 time points). In addition, a high-resolution structural image was acquired in using FSPGR sequence (BRAVO) with parameters (TR = 12.23 ms, TE = 5.18 ms, inversion time [TI] = 450 ms and flip angle = 12°) with an isotropic 1 mm voxel; field of view: 256 × 256.

### Quality control

Regarding head motion during image acquisition, we used the method of scrubbing within regression (spike regression) suggested by Yan et al. to minimize the effect of head motion on FC measurement [30]. This method identifies “bad” time points using a threshold of framewise displacement (FD) > 0.2 mm as well as one back and two forward neighbors [31]; each “bad” time point was modeled as a separate regressor in the regression models [32, 33]. In addition, the parameters of motion correction did not differ between groups.

### Functional connectivity analysis

Separate rs-fMRI analyses were performed for the two clinical trials. All preprocessing was performed using the Data Processing Assistant for Resting-State fMRI (http://www.restfmri.net), which is based on Statistical Parametric Mapping (http://www.fil.ion.ucl.ac.uk/spm) and the Resting-State fMRI Data Analysis Toolkit (http://www.restfmri.net). The functional scans received slice-timing correction, motion correction, and were normalized to a standard anatomical space (Montreal Neurological Institute). Additional preprocessing steps were used to prepare the data for FC analysis. These were as follows: 1) spatial smoothing using a Gaussian kernel (6-mm full width at half-maximum), 2) temporal filtering (0.009 Hz < f < 0.08 Hz), and 3) removal of spurious or nonspecific sources of variance through regression of the following variables. (a) Six head motion parameters and autoregressive models of motion: six head motion parameters, six head motion parameters one time point before, and the 12 corresponding squared items [34] (Friston 24-parameter model); (b) the mean whole-brain signal; (c) the mean signal within the lateral ventricles; and (d) the mean signal within a white matter mask. The regressors used in temporal signal censoring through regression where scrubbing was performed simultaneously to minimize the effect of head motion on the measurement of FC [33, 35]. The seed-based FC analyses were computed in left/right side thalamus according to the automated anatomical labeling (AAL) atlas [36, 37]. In order to improve the data normality for t tests, Fisher’s r-to-z transformation was used to convert correlation maps into z maps [38, 39]. The z-transformed maps of these participants at baseline and 3 days postinfusion were evaluated by statistical models. Furthermore, group-level analyses for the effect of group (ketamine vs. placebo)-by-time (baseline vs. postinfusion) interaction on each seed region-to-whole-brain FC were performed by 3dLME of AFNI 23.0.07 [40]. In the models, treatment and time were regarded as the two independent variables. Treatment was coded as either active treatment (ketamine) or placebo (normal saline or midazolam), and time was coded as either study baseline or postinfusion. In clinical trial 1, 0.2 mg/kg and 0.5 mg/kg ketamine infusions were combined into a single ketamine infusion group in the neuroimaging analyses because of the similar clinical outcomes between groups (symptom reduction rate: total HDRS: 42.6% in the 0.2 mg/kg group vs. 42.6% in the 0.5 mg/kg group, *p* = 0.994; total MADRS: 37.1% in the 0.2 mg/kg group vs. 32.6% in the 0.5 mg/kg group, *p* = 0.652). Age and sex and education were added as covariates of no interests. We focused on the interaction effect of treatment and time and adopted a cluster-forming threshold of *p*  <  0.005 (uncorrected), Monte-Carlo simulation with 10,000 times in AFNI (3dClustSim) presented a minimum cluster size of 58 voxels with a significance threshold at *p*  <  0.05 that correct for false positive rates were reported. To further understand ketamine’s impact on FCs, we calculated the FCs of each participant and performed post-hoc testing. FDR adjusted for multiple comparisons was performed between baseline and 3 days postinfusion separately by groups. Thalamocortical connectivity was computed by using a well-defined seed of AAL atlas and the statistical map was subsequently displayed in probabilistic atlases of the same dimension that were derived from structural segmentations provided by the Harvard Center [37]. With regard to the FCs with significant interaction effects of treatment and time, and in order to reduce the bias from the outliers [41], we used spearman rank partial correlations to investigate the associations between the changes in the FCs and the changes in the depressive and suicidal symptoms with age and sex as covariates. In addition, we also assessed the associations between the changes in the FCs and the changes in the infusion-related dissociation symptoms with age and sex as covariates. The FCs with *p* < 0.05 (uncorrected for multiple comparisons) were reported first. Furthermore, regarding the correction for multiple comparisons in the association analyses (4 clinical measures and 18 significant interactions in clinical trial 1; 6 clinical measures and 4 significant interactions in clinical trial 2), the *p*-values for the significances in the association analyses were revised to 0.0007 (0.05/72) and 0.002 (0.05/24) in clinical trials 1 and 2, respectively [42].

### Statistical analysis for clinical data

We used independent t-test for continuous variables and Fisher’s chi-square tests for nominal variables to assess the differences of demographic and clinical data between clinical trials. A value of *p* < 0.05 was used to indicate statistical significance.

## Results

Table 1 showed the demographic characteristics and clinical symptoms among patients with TRD in clinical trials 1 and 2. Patients in clinical trial 1 were significantly older than those in clinical trial 2 (*p* < 0.001), with a similar sex distribution (*p* = 0.823), BMI (*p* = 0.272), and duration of illness (*p* = 0.481) (Table 1). The treatment refractoriness, measured by MSM, was significantly higher (*p* < 0.001) in patients in clinical trial 2 than those in clinical trial 1 (Table 1). Patients in clinical trial 2 had a higher prevalence (*p* < 0.001) of any suicide attempt, panic disorder comorbidity (*p* = 0.007), and generalized anxiety disorder comorbidity (*p* = 0.009) compared with those in clinical trial 1 (Table 1). Patients with TRD were scored significantly higher on the MADRS (*p* < 0.001; *p* = 0.011), MADRS item 10 (*p* < 0.001; *p* < 0.001), and HDRS item 3 (*p* < 0.001; *p* < 0.001) at baseline and at Day 3 postinfusion compared with those in clinical trial 2 (Table 1). Supplementary table 2 showed no differences in medication use patterns between groups in clinical trials 1 and 2. However, a higher rate (62.5% vs. 35.4%, *p* = 0.014) of prescription of atypical antipsychotics was noted in clinical trial 1 than in clinical trial 2. Supplementary Table 3 showed that the normal saline group did not statistically differ from the ketamine infusion group on any of the clinical measures, including the HDRS (43% vs. 30%, *p* = 0.094) and MADRS (34% vs. 24%, *p* = 0.196) reductions, indicating antidepressant effects in clinical trial 1. In clinical trial 2, patients with 0.5 mg/kg ketamine had greater reduction rates on total MADRS scores (33% vs. 15%, *p* = 0.022), HDRS item 3 scores (51% vs. 28%, *p* = 0.040), and MADRS item 10 scores (48% vs. 26%, *p* = 0.031) at Day 3 compared with those with 0.045 mg/kg midazolam. However, the changes at Day 3 in the total HDRS scores (*p* = 0.068), PANSI-PI scores (*p* = 0.124), and PANSI-NSI scores (*p* = 0.100) did not differ between the ketamine and midazolam groups.

**Table 1:** Baseline demographic and clinical characteristics between two clinical trials.

	Clinical trial 1 (*n* = 48)	Clinical trial 2 (*n* = 48)	*p*-value
Enrolled definition	TRD	TRD-SI	
Age (years, SD)	46.15 (10.67)	32.35 (11.07)	<0.001
Female (n, %)	35 (72.9)	33 (68.8)	0.823
Infusion group (n, %)			
Ketamine 0.5 mg/kg	16 (33.3)	24 (50.0)	
Ketamine 0.2 mg/kg	16 (33.3)	–	
Normal saline	16 (33.3)	–	
Midazolam 0.045 mg/kg	–	24 (50.0)	
BMI (SD)	23.80 (5.04)	25.06 (6.06)	0.272
Education (years, SD)	12.44 (3.38)	14.54 (2.33)	0.001
Duration of illness (years, SD)	10.96 (8.32)	9.79 (7.84)	0.481
History of suicidal attempt (*n*, %)	21 (43.8)	45 (93.8)	<0.001
MSM scores (SD)	8.19 (1.57)	9.65 (2.01)	<0.001
Clinical symptoms at baseline (SD)			
HDRS	21.85 (4.76)	22.29 (4.09)	0.630
HDRS item 3	1.71 (0.85)	2.67 (0.52)	<0.001
MADRS	33.17 (6.94)	37.56 (4.64)	<0.001
MADRS item 10	2.65 (1.35)	4.29 (0.46)	<0.001
PANSI-PI		11.50 (4.51)	
PANSI-NSI		30.35 (6.95)	
Clinical symptoms at Day 3 (SD)			
HDRS	13.75 (7.01)	16.57 (6.62)	0.046
HDRS item 3	0.83 (0.83)	1.65 (1.10)	<0.001
MADRS	23.10 (10.54)	28.83 (10.96)	0.011
MADRS item 10	1.27 (1.16)	2.71 (1.50)	<0.001
PANSI-PI		13.04 (5.26)	
PANSI-NSI		24.83 (10.12)	
Psychiatric comorbidities (*n*, %)			
PTSD	9 (18.8)	14 (29.2)	0.339
Panic disorder	20 (41.7)	34 (70.8)	0.007
Generalized anxiety disorder	30 (62.5)	42 (87.5)	0.009

*TRD* treatment-resistant depression, *SI* suicidal ideation, *SD* standard deviation, *BMI* body mass index, *MADRS* Montgomery-Asberg Depression Rating Scale, *HDRS* Hamilton Depression Rating Scale, *PTSD* post-traumatic stress disorder, *PANSI* Positive and Negative Suicide Ideation Inventory, *PI* Positive Ideation, *NSI* Negative Suicide Ideation, *MSM* Maudsley Staging Method.

**Fig. 1:**
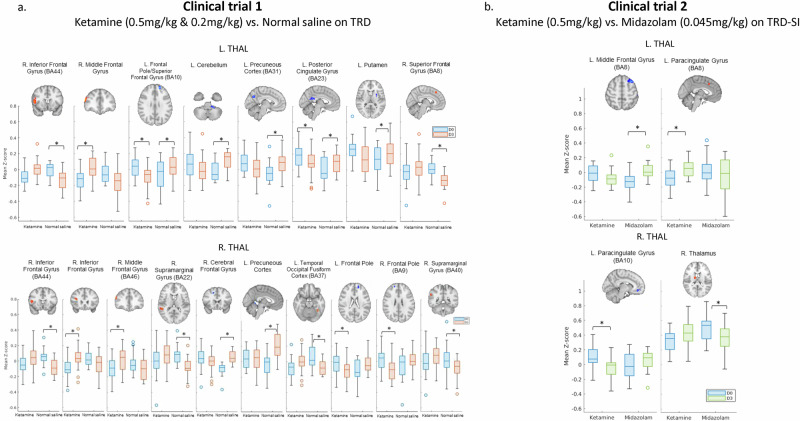
Functional connectivity changes between infusion groups in clinical trials 1 and 2. **a** Ketamine significantly decreased the thalamocortical connectivities between the left thalamus and left frontal pole (BA10) as well as the left posterior cingulate gyrus (BA23), whereas normal saline significantly increased these thalamocortical connectivities; **b** Compared to midazolam, ketamine increased the thalamocortical connectivity between the left thalamus and left anterior paracingulate gyrus (BA8), but decreased the thalamocortical connectivity between the right thalamus and left rostral paracingulate gyrus (BA10). TRD treatment-resistant depression, SI suicidal ideation, L. left, R. right, Thal thalamus.

Table 2 and Fig. 1 revealed the effects of group-by-time interaction on thalamocortical connectivities between two clinical trials. In clinical trial 1, the significance of group-by-time interaction was observed in the FCs of left thalamus with right inferior frontal cortex (BA44), right middle frontal cortex, left frontal pole (BA10), left cerebellum, left precuneous cortex (BA31), left posterior cingulate cortex (BA23), left putamen, and right superior frontal cortex (BA8), and noted in the FCs of right thalamus with right inferior frontal cortex (BA44), right middle frontal cortex (BA46), right supramarginal cortex, left precuneous cortex, left temporal occipital fusiform cortex (BA37), and bilateral frontal poles (Table 2). In clinical trial 2, we only found the group-by-time interaction significance in FCs between left thalamus and left middle frontal cortex (BA8) as well as left paracingulate cortex (BA8) and between right thalamus and left paracingulate cortex (BA10) (Table 2).

**Table 2:** The effects of group-by-time interaction on thalamocortical functional connections between two clinical trials.

Clinical trial 1		Cluster	Coordinate			(*p* < 0.005)	
Effect	Seed	Size	x	y	z	Z	Harvard–Oxford Structure Atlas
Group x	L. Thal	313	50	14	8	4.16	R. Inferior Frontal Gyrus (BA44)
Time		152	54	38	20	4.74	R. Middle Frontal Gyrus
		143	−18	52	28	−4.59	L. Frontal Pole/Superior Frontal Gyrus (BA10)
		92	−22	−38	−44	−3.68	L. Cerebellum
		92	−6	−66	34	−4.20	L. Precuneous Cortex (BA31)
		90	−4	−46	22	−3.87	L. Posterior Cingulate Gyrus (BA23)
		70	−26	6	10	−3.28	L. Putamen
		59	6	30	48	3.93	R. Superior Frontal Gyrus (BA8)
	R. Thal	148	48	16	10	3.77	R. Inferior Frontal Gyrus (BA44)
		99	34	24	18	3.78	R. Inferior Frontal Gyrus
		95	54	36	24	4.40	R. Middle Frontal Gyrus (BA46)
		79	52	−40	12	4.02	R. Supramarginal Gyrus (BA22)
		74	16	20	44	−3.76	R. Cerebral Frontal Gyrus
		65	−2	−50	0	−4.05	L. Precuneous Cortex
		61	−36	−50	−18	4.08	L. Temporal Occipital Fusiform Cortex (BA37)
		59	−16	50	22	−3.53	L. Frontal Pole
		58	18	52	26	−3.58	R. Frontal Pole (BA9)
		58	58	−44	32	3.54	R. Supramarginal Gyrus (BA40)

Clinical trial 2		Cluster	Coordinate			(*p* < 0.005)	
Effect	Seed	Size	x	y	z	Z	Harvard–Oxford Structure Atlas
Group x	L. Thal	117	−32	22	54	−4.22	L. Middle Frontal Gyrus (BA8)
Time		58	−2	20	44	3.36	L. Paracingulate Gyrus (BA8)
	R. Thal	148	−8	42	−10	−3.55	L. Paracingulate Gyrus (BA10)
		138	12	−6	16	3.88	R. Thalamus

*L* left, *R* Right, *Thal* Thalamus.

In clinical trial 1, ketamine significantly decreased the thalamocortical connectivities between the left thalamus and left frontal pole (BA10) as well as the left posterior cingulate gyrus (BA23), whereas normal saline significantly increased these thalamocortical connectivities (Fig. 1a). Additionally, compared to normal saline, ketamine increased the thalamocortical connectivities between the bilateral thalamus and right middle frontal cortex and between the right thalamus and right inferior frontal cortex (Fig. 1a). In clinical trial 2, compared to midazolam, ketamine increased the thalamocortical connectivity between the left thalamus and left anterior paracingulate gyrus (BA8), but decreased the thalamocortical connectivity between the right thalamus and left rostral paracingulate gyrus (BA10) (Fig. 1b). However, midazolam increased the thalamocortical connectivity between the left thalamus and left middle frontal cortex compared to ketamine in clinical trial 2 (Fig. 1b).

Finally, regarding the multiple comparisons of the correlation analyses with the revised *p*-values of 0.0007 (0.05/72) and 0.002 (0.05/24) in clinical trial 1 and trial 2, we found no significant associations between changes in thalamocortical FC and changes in clinical measures, including HDRS, MADRS, and PANSI. However, it may be interesting to note that patients receiving a single ketamine infusion in clinical trial 1 showed a non-significantly positive correlation (r = 0.038, *p* = 0.030) between the change in MADRS scores and the change in the FC between the right thalamus and right middle frontal cortex (BA46) (Supplementary Fig. 3a); patients receiving a single infusion of ketamine in clinical trial 2 showed a non-significantly negative correlation (r = −0.045, *p* = 0.043) between the change in PANSI-PI scores and the change in the FC between the right thalamus and left rostral paracingulate cortex (BA10) (Supplementary Fig. 3b). In addition, we found no associations between the changes in the FCs and the changes in the infusion-related dissociation symptoms (all *p* > 0.05).

## Discussion

Our study aimed to clarify the role of thalamocortical connectivity in the antidepressant and antisuicidal effects of ketamine treatment among patients with TRD. We discovered that low-dose ketamine infusion may result in certain alterations in thalamocortical connectivity, like increased FCs between the bilateral thalamus and right middle frontal cortex (BA46) and between the left thalamus and left anterior paracingulate gyrus (BA8), and decreased FCs between the right thalamus and bilateral frontal pole (BA9) and between the right thalamus and left rostral paracingulate gyrus (BA10). Furthermore, we found no association between changes in thalamocortical FC and changes in clinical symptoms in the correlation analyses with the multiple comparison correction.

Decreased FC between the thalamus and bilateral middle frontal and prefrontal regions is a TRD biomarker [9, 10]. The modulation of this thalamocortical connectivity may be key to the antidepressant effect of NMDAR antagonists, including ketamine [16]. Our results revealed ketamine-related increases in FC between the bilateral thalamus and the right middle frontal cortex (BA46) and between the left thalamus and left anterior paracingulate gyrus (BA8), which may support the hypofrontality of TRD, particularly regarding the dorsal and lateral parts of the PFC (DLPFC) and dorsal anterior cingulate cortex [43]. Our previous [^18^F]fluorodeoxyglucose positron emission tomography study also identified increases in the glucose metabolism of the PFC and dorsal anterior cingulate cortex among patients with TRD who received a single infusion of 0.5 mg/kg or 0.2 mg/kg ketamine [26].

We identified reduced thalamocortical connectivity with the left posterior cingulate cortex (BA23), left rostral paracingulate gyrus (BA10), and bilateral frontal poles (BA9) after a single ketamine infusion. These three regions are important hubs of the default mode network (DMN), which is key to the pathomechanisms of major depressive disorder and TRD [44–46]. Li et al. identified two default mode subnetworks in patients with depression and healthy controls and reported that the signals with the highest amplitude in the anterior subnetwork were in the medial PFC, and that those with the highest amplitude in the posterior network were in the bilateral precuneus/posterior cingulate cortex [44]. They identified increased FC within both the anterior and posterior subnetworks of the DMN in patients with depression, and their results suggested that 12-week antidepressant treatment normalized the posterior, but not the anterior, subnetwork [44].

The thalamus has been shown to be structurally and functionally connected to DMN regions [47]. Evidence further suggested that the anterior and mediodorsal thalamus may belong to the DMN [47]. Altered DMN is one of the core pathomechanisms underlying TRD [47, 48]. A systematic review of neuroimaging studies showed that TRD was associated with reduced FCs within the DMN, reduced FCs between components (i.e., parahippocampal cortex) of the DMN and several frontal regions (i.e., medial frontal cortex, DLPFC), and increased FCs between middle temporal cortex and superior frontal cortex [48]. Runia et al additionally reported increased resting-state activity, measured by the fractional amplitude of low-frequency fluctuations, in the thalamus, anterior cingulate cortex and medial frontal cortex among patients with TRD [48]. Scheidegger et al. suggested that the rapid antidepressant effect of ketamine treatment may be associated with ketamine-related decreased FC of the DMN with the pregenual anterior cingulate cortex and medial prefrontal cortex through the posterior cingulate cortex [49]. Zacharias et al. demonstrated that after ketamine administration, patients with TRD had decreased FC in the medial prefrontal cortex using the seed (posterior cingulate cortex /precuneus area)-based DMN FC analysis [50].

In our study, we found decreased FCs between the right thalamus and bilateral frontal pole (BA9) and between the right thalamus and left rostral paracingulate gyrus (BA10), which also may echo the findings of Can et al. [51]. Can and colleagues discovered that ketamine decreased anterior DMN connectivity among patients with chronic suicidality [51]. Conversely, the placebo infusion exerted opposite effects on the anterior (left frontal pole) and posterior (posterior cingulate cortex) subnetworks of the DMN to those caused by ketamine infusion, which may support the lack of rapid antidepressant and antisuicidal effects of placebo treatment. Increasing evidence has reported that DMN suppression is functionally important for the successful operation of cognitive processes, which played a crucial role in the antidepressant effect of ketamine infusion [52, 53] and was compatible with the findings of our current (decreased anterior DMN connectivity) and past (improved cognitive function) studies [54]. As mentioned, we found no such effect of the DMN suppression in the control group. Furthermore, FC changes, such as reduced FCs between the bilateral thalamus and inferior frontal cortex (BA44) and within the thalamus and elevated FCs between the bilateral thalamus and left precuneus cortex (BA31), were associated with the effect of the placebo infusion in our study. Previous studies indicated that the salience network FCs (i.e., dorsal anterior cingulate cortex, anterior insula and inferior frontal cortex), but not the DMN FCs, were linked to clinical placebo responses among patients with major depressive disorder [55, 56]. Schienle et al found that the placebo reduced FCs in the fronto-cognitive control network, including inferior frontal cortex and lateral PFC [57]. Kong et al. discovered associations between the placebo effect and the bilateral lateral/orbital PFC and right fusiform cortex [58].

Finally, a positive correlation (*p* = 0.030) between the change in depressive symptoms and the change in the FC between the right thalamus and right middle frontal cortex in clinical trial 1 and a negative correlation (*p* = 0.043) between the change in PANSI-PI scores and the change in the FC between the right thalamus and left rostral paracingulate cortex in clinical trial 2 did not survive the statistical significance after multiple comparison corrections (p-values: 0.0007 (0.05/72) and 0.002 (0.05/24) in clinical trial 1 and trial 2, respectively), which may imply that our findings of associations between thalamocortical FC changes and ketamine’s antidepressant and antisuicidal effects may be a coincidence. Although our study showed thalamocortical FC changes at post-ketamine infusion but failed to support the associations of such FC changes with ketamine’s antidepressant and antisuicidal effects, whether other FCs beyond the thalamocortical circuit may serve a crucial role in ketamine-related antidepressant and antisuicidal effects would need further research.

This study has several limitations. First, the medications of the patients with TRD were continued and unchanged during the ketamine treatment and MRI; therefore, the current study findings can be explained as being related to the add-on effects of a low-dose ketamine infusion. The changes in FCs may have resulted from combined regulatory effects of ketamine and the medications that the patients were already taking. An add-on study design is ethically appropriate for patients with TRD, particularly for those with strong SI, and may provide more naturalistic data. Second, the placebo used was normal saline in clinical trial 1 and midazolam in clinical trial 2, which may partially explain why more differences in FCs existed between the groups in clinical trial 1 than between the groups in clinical trial 2. For example, Wang et al. reported that, compared with normal saline infusion, 0.03 mg/kg midazolam infusion reduced the FCs in the attention- and executive function-related regions (i.e., medial PFC, superior frontal cortex) but increased the FCs in the sensorimotor regions (i.e., postcentral and precentral cortex) [39]. Furthermore, the discrepancies in FCs between the groups may have been caused by differences in illness severity and age between the research groups. Age and disease severity were greater in clinical trial 2 than in clinical trial 1. Brown et al. found that increased thalamo-temporal FC was associated with the severity of depression [59]. However, comparing one clinical trial with saline and another with midazolam may not seem fully appropriate. Further studies using the same placebo would be required to validate our results. Third, though we identified significant group x time interaction effects for thalamocortical FC, we found no significant symptom improvement in the ketamine group compared with the normal saline group in clinical trial 1. Therefore, it may not make sense to investigate in this sample associations with symptom improvement, as those can at least in part be attributed to a placebo effect. Whether the changes in the thalamocortical FC may be associated with ketamine’s or placebo’s effect would need further investigation. Forth, the small sample sizes of our two clinical trials may limit the generalization of our findings and may be related to the condition that there were no overlapping clusters between clinical trials 1 and 2. Marek et al further suggested that reproducible brain-wide association studies may require thousands of individuals [60]. A future study with a large sample size would be necessary to validate our findings.

In conclusion, a single infusion of low-dose ketamine changed thalamocortical connectivity, including the increase in FCs between the thalamus and middle frontal cortex (BA46) and between the thalamus and anterior paracingulate gyrus (BA8) and the decrease in FC between the thalamus and rostral paracingulate gyrus (BA10) and the frontal pole, among patients with TRD. Our findings may inspire clinicians and researchers to clarify whether patients with profound thalamocortical dysconnectivity may be more suitable for low-dose ketamine treatment compared with those without. Further studies are necessary to improve the generalizability of our findings regarding the sustained antidepressant and antisuicidal effects of repeated ketamine infusions.

## Supplementary information

Supplementary Tables

Supplementary figure

## Data Availability

The data that support the findings of this study are available from the corresponding author, MHC, upon reasonable request.
