# Supplementary material for: Thalamocortical functional connectivity and rapid antidepressant and antisuicidal effects of low-dose ketamine infusion among patients with treatment-resistant depression
Source: Mol Psychiatry. 2024 Jul 6;30(1):61–8. doi: 10.1038/s41380-024-02640-3 (PMC11649554; doi:10.1038/s41380-024-02640-3)
Supplement: Supplementary file 1 — Supplementary Tables [file 41380_2024_2640_MOESM1_ESM.docx]

Supplementary table 1. Demographic and clinical characteristics between groups in two clinical trials.

|  | Clinical trial 1  (n = 48) | | Clinical trial 2  (n = 48) | | |
| --- | --- | --- | --- | --- | --- |
|  | Ketamine 0.5 or 0.2mg/kg  (n = 32) | Normal saline  (n = 16) | Ketamine 0.5mg/kg  (n = 24) | | Midazolam 0.045mg/kg  (n = 24) |
| Age (years, SD) | 44.31 (11.43) | 49.81 (8.12) | 30.58 (11.03) | | 34.13 (11.05) |
| Sex (n, %) |  |  |  | |  |
| Male | 7 (21.9) | 6 (37.5) | 9 (37.5) | | 6 (25.0) |
| Female | 25 (78.1) | 10 (62.5) | 15 (62.5) | | 18 (75.0) |
| BMI (SD) | 22.93 (3.93) | 24.49 (5.47) | 25.57 (6.54) | | 24.54 (5.64) |
| Education (years, SD) | 12.63 (3.54) | 12.06 (3.11) | 14.04 (2.53) | | 15.04 (2.05) |
| Duration of illness (years, SD) | 11.41 (9.38) | 10.06 (5.83) | 8.79 (7.21) | | 10.79 (8.47) |
| History of suicidal attempt (n, %) | 14 (43.8) | 7 (43.8) | 18 (75.0) | | 20 (83.3) |
| MSM scores (SD) | 8.13 (1.57) | 8.44 (1.55) | 9.17 (1.99) | | 10.13 (1.94) |
| Clinical symptoms at baseline (SD) |  |  |  | |  |
| HDRS | 21.59 (5.05) | 22.56 (3.83) | 21.54 (4.36) | | 23.04 (3.74) |
| HDRS item 3 | 1.69 (0.93) | 1.75 (0.68) | 2.67 (0.57) | | 2.67 (0.48) |
| MADRS | 32.63 (8.04) | 34.00 (4.71) | 36.04 (4.49) | | 39.08 (4.03) |
| MADRS item 10 | 2.66 (1.38) | 2.63 (1.31) | 4.21 (0.42) | | 4.38 (0.50) |
| PANSI-PI | - | - | 11.58 (5.09) | | 11.42 (3.96) |
| PANSI-NSI | - | - | 30.58 (7.41) | | 30.13 (6.12) |
| Psychiatric comorbidities (n, %) |  |  |  | |  |
| PTSD | 4 (12.5) | 5 (31.3) | | 8 (33.3) | 6 (25.0) |
| Panic disorder | 15 (46.9) | 5 (31.3) | | 17 (70.8) | 17 (70.8) |
| Generalized anxiety disorder | 20 (62.5) | 10 (62.5) | | 20 (83.3) | 22 (91.7) |

SD: standard deviation; BMI: body mass index; MSM: Maudsley Staging Method; MADRS: Montgomery-Asberg Depression Rating Scale; HDRS: Hamilton Depression Rating Scale; PANSI: Positive and Negative Suicide Ideation Inventory; PI: Positive Ideation; NSI: Negative Suicide Ideation; PTSD: post-traumatic stress disorder.

Supplementary table 2. Oral medications between groups in clinical trials 1 and 2.

|  | Clinical trial 1  (n = 48) | | Clinical trial 2  (n = 48) | |
| --- | --- | --- | --- | --- |
|  | Ketamine 0.5 or 0.2mg/kg  (n = 32) | Normal saline  (n = 16) | Ketamine 0.5mg/kg  (n = 24) | Midazolam 0.045mg/kg  (n = 24) |
| Any antidepressant | 32 (100.0) | 16 (100.0) | 23 (95.8) | 24 (100.0) |
| p-value (within trial) | >0.999 | | >0999 | |
| p-value (between trial) | >0999 | | | |
| Combined antidepressants | 13 (40.6) | 9 (56.3) | 9 (37.5) | 9 (37.5) |
| p-value (within trial) | 0.366 | |  | |
| p-value (between trial) | 0.535 | | | |
| Mood stabilizers | 7 (21.9) | 3 (18.8) | 4 (16.7) | 4 (16.7) |
| p-value (within trial) | >0.999 | | >0.999 | |
| p-value (between trial) | 0.794 | | | |
| Atypical antipsychotics | 22 (68.8) | 8 (50.0) | 11 (45.8) | 6 (25.0) |
| p-value (within trial) | 0.226 | |  | |
| p-value (between trial) | **0.014** | | | |

Supplementary table 3. Symptom changes between groups in clinical trials 1 and 2.

|  | Clinical trial 1  (n = 48) | | | Clinical trial 2  (n = 48) | |
| --- | --- | --- | --- | --- | --- |
|  | Ketamine 0.5 or 0.2mg/kg  (n = 32) | Normal saline  (n = 16) | | Ketamine 0.5mg/kg  (n = 24) | Midazolam 0.045mg/kg  (n = 24) |
| △(D3-D1)/D1 |  |  | |  |  |
| △HDRS | -0.43 (0.27) | -0.30 (0.24) | | -0.33 (0.28) | -0.19 (0.24) |
| p-value | 0.094 | | | 0.068 | |
| △HDRS item 3 | -0.52 (0.44) | -0.40 (0.46) | | -0.51 (0.40) | -0.28 (0.35) |
| p-value | 0.383 | | | **0.040** | |
| △MADRS | -0.34 (0.30) | -0.24 (0.19) | | -0.33 (0.28) | -0.15 (0.22) |
| p-value | 0.196 | | | **0.022** | |
| △MADRS item 10 | -0.54 (0.38) | -0.34 (0.38) | | -0.48 (0.35) | -0.26 (0.32) |
| p-value | 0.100 | | | **0.031** | |
| △PANSI-PI | **-** | | | 0.25 (0.32) | 0.09 (0.36) |
| p-value | **-** | | | 0.124 | |
| △PANSI-NSI | **-** | | | -0.25 (0.27) | -0.12 (0.26) |
| p-value | **-** | | | 0.100 | |
| Changes in the dissociative symptoms between 40-min infusion and baseline# (SD) |  | | |  | |
| BPRS positive symptom subscale | 0.08 (0.41) | | 0.00 (0.00) | - |  |
| p-value | 0.323 | | | - | |
| CADSS | **-** | | **-** | 5.88 (3.30) | 1.38 (3.95) |
| p-value |  | | | **<0.001** | |

MADRS: Montgomery-Asberg Depression Rating Scale; HDRS: Hamilton Depression Rating Scale; PANSI: Positive and Negative Suicide Ideation Inventory; PI: Positive Ideation; NSI: Negative Suicide Ideation; BPRS: Brief Psychiatric Rating Scale; CADSS: Clinician-Administered Dissociative States Scale

#: changes were defended by (scores at 40-min infusion minus scores at baseline); dissociation symptoms were measured by the Brief Psychiatric Rating Scale positive subscale in clinical trial 1 and the Clinician-Administered Dissociative States Scale in clinical trial 2.
