# Supplementary material for: Thalamocortical functional connectivity and rapid antidepressant and antisuicidal effects of low-dose ketamine infusion among patients with treatment-resistant depression
Source: Mol Psychiatry. 2024 Jul 6;30(1):61–8. doi: 10.1038/s41380-024-02640-3 (PMC11649554; doi:10.1038/s41380-024-02640-3)

Supplementary figure 1. Flowchart of clinical trial 1

Supplementary figure 2. Flowchart of clinical trial 2.


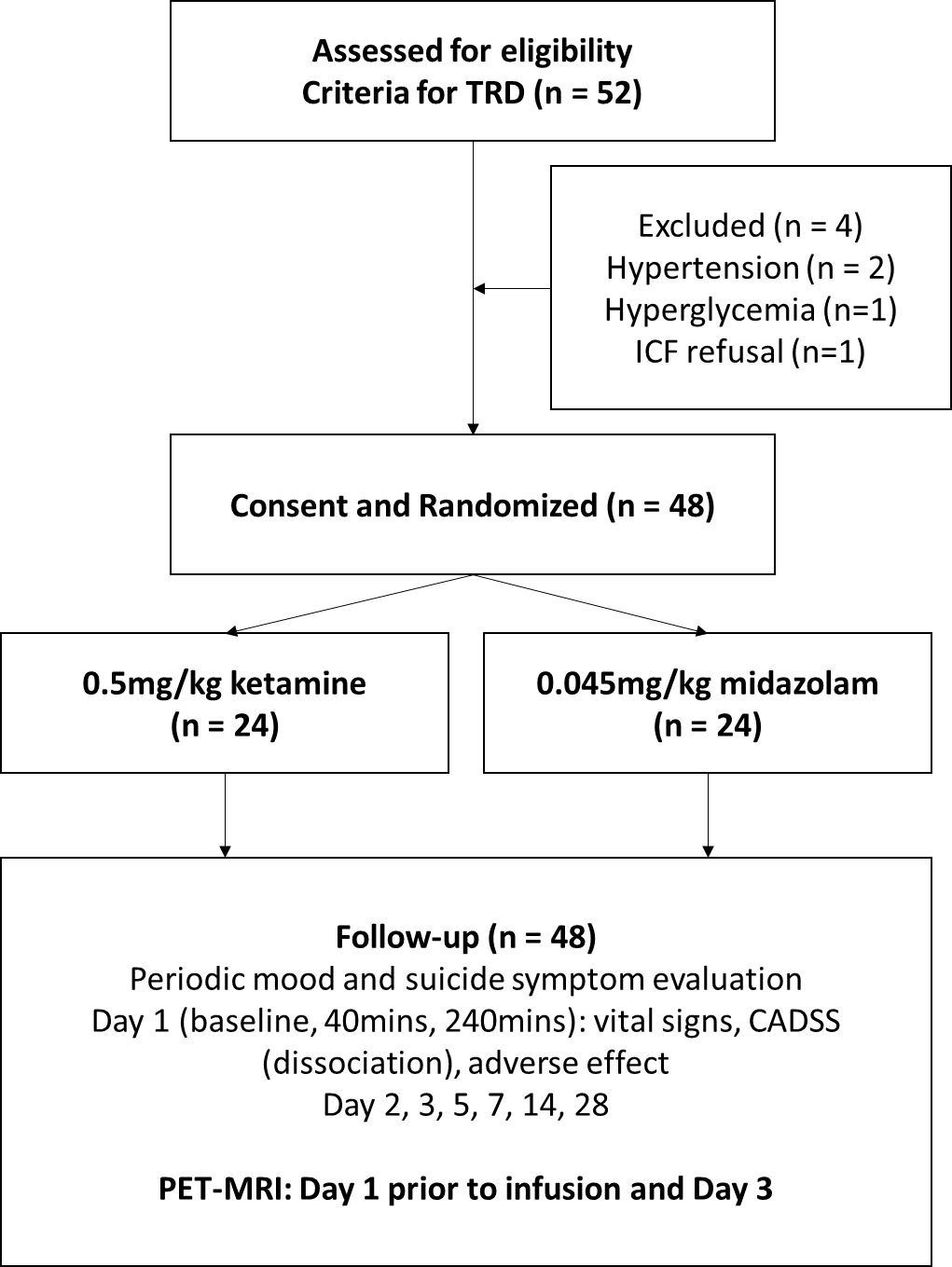


Supplementary figure 3. Correlations analyses between FC changes and symptom changes in clinical trials 1 and 2. (a) Increases in FC between right thalamus and right middle frontal cortex (BA46) were associated with less improvement on the MADRS scores; (b) Decreases in FC between right thalamus and left paracingulate cortex (BA10) were associated with more improvement on the PANSI-PI scores.


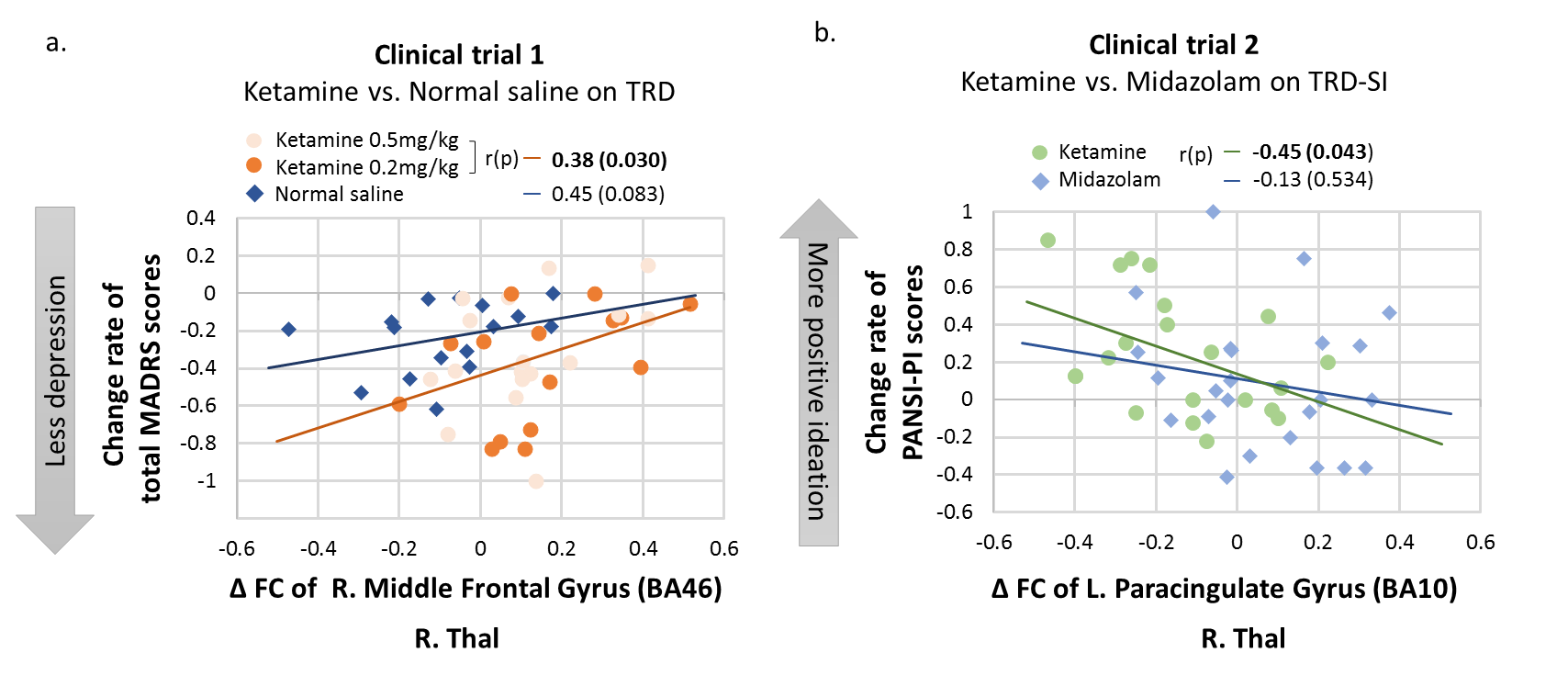

Supplement: Supplementary file 2 — Supplementary figure [file 41380_2024_2640_MOESM2_ESM.doc]
